# Knowledge and Experience of Neonatal Resuscitation among Midwives in Tamale

**DOI:** 10.1155/2019/3652608

**Published:** 2019-01-02

**Authors:** Afizu Alhassan, Abdul-Ganiyu Fuseini, Wahab Osman, Alhassan Basour Adam

**Affiliations:** ^1^Senior Health Tutor, Nursing and Midwifery Training College, Kpembe, P.O. Box SL98, Salaga, Ghana; ^2^Department of Nursing, School of Allied Health Sciences, University for Development Studies, P.O. Box 1883, Tamale, Ghana; ^3^In-Service Training Coordinator, Tamale Teaching Hospital, P.O. Box 16, Tamale, Ghana; ^4^Senior Health Tutor, Bolga Midwifery Training College, P.O. Box 255, Bolgatanga, Ghana

## Abstract

*Background. *Efforts to reduce under-five mortality across the globe are being hindered by a disproportionately high rate of neonatal deaths. About a quarter of these neonatal deaths are caused by birth asphyxia. Available evidence shows that effective neonatal resuscitation delivered by providers skilled in and knowledgeable about neonatal resuscitation can significantly reduce neonatal mortality rates.* Objectives. *This cross-sectional study was conducted to determine knowledge and experience in neonatal resuscitation among midwives in Tamale, and the factors associated with their knowledge on neonatal resuscitation.* Methods. *This was a cross-sectional study involving all midwives practicing in three large hospitals in Tamale. We developed a questionnaire to collect data on demographic characteristics of participants, and their knowledge and experience in neonatal resuscitation. We used the Statistical Package for Social Sciences (SPSS), version 21, to analyse the data. Demographic characteristics of participants were summarized using descriptive statistics. Pearson's correlation was used to determine associations between knowledge and some selected demographic features, while the one-way ANOVA was conducted to determine differences in level of knowledge based on the demographic features.* Results.* 98.1% of the participants in this study had insufficient knowledge on neonatal resuscitation. Midwives at the Tamale Central Hospital demonstrated a statistically significantly higher level of knowledge* (24.67 ± 2.79, p = .014)*, compared to those at the Tamale Teaching Hospital* (22.92 ± 4.56, p = .028) *and Tamale West Hospital* (21.50 ± 6.24, p = .021)*. Those who had a first-degree qualification in midwifery and those with a Post-NAC/NAP midwifery certificate had a statistically significantly higher knowledge than those with a diploma in midwifery. Training in neonatal resuscitation was associated with more knowledge in neonatal resuscitation (*r*(158) = .195, p = .013). In terms of experience, 55% of the participants in this study were not experienced in performing neonatal resuscitation. There were no differences in their level of experience based on their academic qualification, work place, and years of practice as a midwife.* Conclusion. *Considering the generally low level of knowledge and experience of midwives in neonatal resuscitation as discovered in this study, there is an urgent need for government to provide more opportunities for all practicing midwives to be trained in this important lifesaving skill.

## 1. Background to the Study

Efforts to meet the target of Millennium Development Goal Four (MDG–4) which was to reduce by two-thirds, between 1990 and 2015, the under-five mortality rate, have produced some positive results in many countries [1]. Deaths of children aged under five years from preventable causes are fewer than ever before. The global under-five mortality rate witnessed a significant decline from 12.7 million to six million deaths a year between 1990 and 2015 [1]. However, this significant drop in child mortality is mostly attributable to a reduction in infant mortality, as neonatal mortality rates have either stagnated or continued to rise [1, 2].

Globally, about 4 million neonatal deaths occur every year, accounting for 43% of under-five child mortality, with 99% of these deaths occurring in low and middle income countries [3–5]. In Ghana, conscious and sustained action to bring down the under-five mortality rate has seen a progressive reduction in deaths from 155 to 60 per 1,000 live births between 1990 and 2014 [1]. Also, although infant mortality remained high in the rest of Africa, infant mortality rate for Ghana dropped from 66 to 41 per 1,000 live births between 1990 and 2014 [1].

However, just like the global situation, this progress in under-five mortality rates seen in Ghana largely reflects improvements in the health of infants and older children. There have not been significant decreases in neonatal death over the period. In fact, neonatal deaths have accounted for a disproportionate number of total under-five deaths [6]. According to UNICEF-Ghana, “In Ghana, a new-born baby dies every 15 minutes, and new-born deaths contribute to 50% of all infants' deaths!” [7, p.1].

Interestingly, neonatal asphyxia has been identified as a major cause of neonatal mortality worldwide. Globally, about 25% of all neonatal deaths are caused by birth asphyxia [8], which is defined by the World Health Organization as the failure to initiate and sustain breathing at birth. In Ghana, about 27% of neonatal deaths are attributed to adverse intrapartum events including birth asphyxia [6, 7]. Meanwhile, birth asphyxia as a cause of neonatal deaths can be effectively treated to a very large extent with timely resuscitation of newborns by healthcare providers who are skilled in and knowledgeable about neonatal resuscitation [8]. Effective resuscitation at birth can prevent up to about 30% of neonatal deaths [9, 10]. A Tanzanian study conducted in 2013 by Msemo et al. [11] revealed that provision of newborn resuscitation, specifically, by skilled birth attendants trained in Helping Babies Breathe (HBB), reduced neonatal mortality by 47%. Competence in neonatal resuscitation among healthcare providers, particularly midwives, is also important because, every year, about 10 million babies require assistance to initiate breathing [10]. In addition, between five percent and ten percent of all babies born in facilities require some degree of resuscitation, such as tactile stimulation, airway clearing, or positioning [12]. Enhancing the competence of midwives in neonatal resuscitation techniques can therefore go a long way to reduce neonatal deaths in many countries. There is the need for data on the knowledge and experience of midwives with regard to neonatal resuscitation to guide government policies on training of midwives in neonatal resuscitation. However, such data is not commonly available in Ghana. The aim of this study, therefore, was to ascertain the knowledge and experience of midwives in neonatal resuscitation, and the factors associated with their knowledge, using Tamale as a case study. The study focused on only midwives because they are the primary birth attendants in all hospitals in Ghana.

### 1.1. Context of Midwifery Training and Certification in Ghana

At present, midwifery training in Ghana is offered in three different ways: the direct diploma, the direct degree, and the post-basic qualification in midwifery. The direct diploma in midwifery is open to all Senior High School graduates with a minimum of six credits in the West African Senior School Certificate Examination (WASSCE) [13]. It is a three-year program run by Nursing and Midwifery Training Colleges in Ghana. These Training Colleges are affiliated to the Kwame Nkrumah University of Science and Technology, and it is the institution that awards the diploma certificates to students after completion [14]. The direct degree in midwifery is also open to all Senior High School graduates, and it has the same entry requirements as the diploma in midwifery, even though different univerisities may have additional criteria. However, that one runs for four years and is run by both public and private universities in Ghana [15, 16].

The post-basic entry is available as either post-diploma or Post-Nurse Assistant-Clinical/Nurse Assistant Preventive (Post NAC/NAP). The Nurse Assistant - Clinical (NAC) and Nurse Assistant - Preventive (NAP) programs are auxiliary nursing programs in Ghana that usually run for two years and products are registered as nurse assistants. After practicing for three years, Nurse Assistants are eligible to enroll in a midwifery training program for two years after which they get a certificate in midwifery [13]. Presently, there is a program in place to upgrade that program to a diploma program so that the point of entry for midwifery in Ghana will be a diploma [17]. For those who already have a diploma in midwifery and wish to upgrade to a degree, the post-diploma entry is available to them. With this, the universities give opportunity for diploma holders to enroll in the degree program at Level 300, and after two years, based on successful completion, they are awarded a Degree in Midwifery [15, 16].

It is important to note that all these midwifery programs are regulated by the Nursing and Midwifery Council of Ghana (N&MC), and candidates are required to pass a licensing exam conducted by the N&MC, after which they are registered as Registered Midwives.

## 2. Methods

### 2.1. Study Design

The study was conducted using a cross-sectional survey with a quantitative approach. In cross-sectional surveys, independent and dependent variables are measured at the same point in time [18].

### 2.2. Study Setting

The study was conducted in Tamale, which is officially called Tamale Metropolitan Area and is the capital town of the Northern Region of Ghana. Tamale is Ghana's fourth largest city with a population of 360,579 people, according to the world population review [19], and is the fastest-growing city in West Africa (Ghanaweb, 2014). The town is located 600 km north of Accra, the capital of Ghana.

There are three major government hospitals in Tamale: Tamale Teaching Hospital, Tamale Central Hospital, and Tamale West Hospital. The Tamale Teaching Hospital is the only tertiary health facility in the northern region of Ghana. It is also the main referral hospital for the three regions of the North, that is, northern, upper east, and upper west regions. According to the 2017 Annual Performance Review report of the hospital, there are 102 midwives working in that hospital [20]. The Tamale Central Hospital was established in July 1929 to cater for the health needs of the northern territory. It is a 186-bed capacity hospital with eight functional wards, and provides a 24-hour service that includes OPD, pharmacy, antenatal care, laboratory service, theatre service, ear, nose, and throat care, and psychiatry. The central hospital has a catchment population of more than 30,000 people within the Tamale metropolis, with monthly outpatient department attendance of about 3,000 cases [21]. There are a total of 80 midwives practicing in that facility. Tamale West Hospital which is currently a referral hospital for the Tamale Metro subdistrict health centres has a capacity of about 180 beds and provides 24-hour services with seven functional wards, namely, Male, Maternity, Labour, Emergency, Children, Female, and Surgical wards [22]. There are about 68 midwives practicing at the Tamale West Hospital. The study was conducted using all these three hospitals as study sites.

### 2.3. Sampling

Owing to the relatively small size of the target population, the researchers adopted the census approach to select the respondents. This means all midwives practicing in the three major government hospitals within the Tamale Metropolis who agreed to participate in the study were involved.

### 2.4. Data Collection Instrument

A structured questionnaire was used to collect data for the study. The questionnaire comprised three main parts designed to capture data about participants' demographic features and their knowledge and experience in neonatal resuscitation. The first part was on participants' demographic features such as age, rank, and academic qualification. The second part was used to capture data about participants' exposure to, or experience in, neonatal resuscitation, including training they had received and actual performance of the task (neonatal resuscitation). This section contained five questions in all.

The third part was designed to measure the knowledge of participants on neonatal resuscitation. It contained a total of 37 multiple choice questions drawn from standard references such as the World Health Organization's Guidelines on Neonatal Resuscitation, American Heart Association's Guidelines for Cardiopulmonary Resuscitation (CPR) and Emergency Cardiovascular Care (ECC) of Paediatric and Neonatal Patients, and a textbook on neonatal resuscitation [8, 12, 23]. Cronbach's alpha for this section of the questionnaire was .796 while overall Cronbach's alpha for the entire questionnaire was .746.

### 2.5. Data Collection Procedure

Following ethical clearance from the participating institutions, the questionnaire, along with a consent form to introduce the study purpose and participant's rights, was personally administered to the participants by the researchers. The questionnaires were administered concurrently in all three study centres by the research team. Once a participant read the consent form and agreed to participate in the study, she was directed to go ahead and complete the attached questionnaire, within 35 minutes, in the presence, and under the supervision, of at least one of the researchers. This was to ensure that participants did not refer to any textbooks or online resources to complete the questionnaire, which could affect the validity of the results. Because of this, there were many times when the researchers had to rearrange convenient times for participants to complete the questionnaire, if a participants could not make time to immediately complete it. Even though participants had 35 minutes to complete the questionnaire, many of them finished earlier than that. Once a participant was done answering, the questionnaire was immediately scanned for completeness, and then it was added to the rest. The data was collected over a period of one month, from 10^th^ July to 10^th^ August, 2018.

### 2.6. Data Management

During data collection, each questionnaire was scanned for completeness immediately after a participant finished answering; those who did not complete any relevant portions were respectfully prompted to complete it. Data entry was done facility by facility. After completing entry of every individual questionnaire, it was marked as entered, so that we did not generate duplicates. When all questionnaires from one facility were completed, they were bagged in an envelope and labelled completed and locked away in a cabinet. Throughout the process of data entry, the data was saved every 10 minutes. At the end of each day, the data was backed up on an external hard drive as well as Microsoft OneDrive online.

### 2.7. Data Analysis

The Statistical Package for Social Sciences, version 21, [24] was used to analyse data from the study. Descriptive statistics such as frequencies, percentages, means, and medians were used to summarize the background characteristics of participants. Experience of midwives on neonatal resuscitation was computed from three main items, that is, type of training received on neonatal resuscitation, number of neonatal resuscitations performed in professional practice, and number of neonatal resuscitations observed. A value of five was allocated for each type of training received in neonatal resuscitation; a value of one for each resuscitation performed; and 0.5 for each resuscitation observed. Therefore, each session of neonatal resuscitation observed contributed only half of a point in neonatal resuscitation experience because observing a procedure does not give you the same experience as performing it yourself. These values were added together to create a scale for neonatal resuscitation experience. From this scale, participants were put into three categories of experience based on their raw scores. The categories were; not experienced (0–16), moderately experienced (17-31), and highly experienced (32 or higher). Descriptive statistics were then used to further summarize the findings.

Knowledge of midwives on neonatal resuscitation was determined using a scale containing 37 multiple choice questions. Seven items were used to assess the knowledge of participants on evaluation of newborns, while 30 items assessed their knowledge on appropriate interventions to carry out. Each correct answer was valued at one point, and a wrong answer attracted no point. Questions that were not answered were treated as wrong answers. A total score on knowledge was calculated for each participant from these 37 items. Participants were then grouped into two categories based on their total score on the knowledge scale: sufficient knowledge (80% or higher) and insufficient knowledge (less than 80%). This categorization was based on previous studies on the subject that used similar benchmarks to categorize knowledge of participants [25–27]. Pearson's correlation was used to determine association between neonatal resuscitation experience and knowledge, knowledge in neonatal resuscitation and previous training on neonatal resuscitation, as well as knowledge and years of practice as a midwife.

The one-way ANOVA was used to determine differences in knowledge of participants based on academic qualification and place of work. We first tested the data on these two variables (place of work and academic qualification) for normality of distribution using Shapiro-Wilk's test, and they were not normally distributed. In addition, we also checked the two variables for homogeneity of variances using Levene's test, and there were significant differences in the variances between the groups. As such, we did not use the classic ANOVA in performing these analyses. Instead, we used Welch's ANOVA. An alpha (P) value of <.05 was considered significant.

### 2.8. Ethical Considerations

We sought approval for the study from the Department of Research and Development, Tamale Teaching Hospital (Approval number: TTH/R&D/SR/113). We also obtained written permission from the Northern Regional Health Directorate, which is in charge of Tamale West and Central Hospitals. Participation in the study was completely voluntary and participants were made aware of their right to refuse to participate in the study. Those who agreed to participate were made to sign a written informed consent. In order to maintain anonymity of participants, the questionnaires, once completed, were immediately added to a larger pool of other completed questionnaires in a random fashion. Confidentiality of all data was also ensured by keeping all questionnaires securely locked in a cabinet.

## 3. Results

### 3.1. Background Characteristics of Participants

Out of a total population of 240 midwives across the three different study sites, it was 160 midwives who eventually participated in the study. The characteristics of that sample are presented in Table 1.

Most of the participants were in their 30s (mean age = 32.94, median 31.00), and had been practicing midwifery on average for about three years (median age of practice = 3.0). Almost half (49.4%) had the least qualification in midwifery in Ghana at present, that is, the Post NAC/NAP Certificate in midwifery.

### 3.2. Knowledge of Midwives on Neonatal Resuscitation

Almost all participants in this study (98.1%) had insufficient knowledge on neonatal resuscitation. The mean score on knowledge was 23.0 while the median was 24.0, with a standard deviation of 4.78. The level of knowledge of participants on neonatal resuscitation is presented in Table 2.

Beyond the general knowledge on neonatal resuscitation as a whole, we also explored further to find out the knowledge of midwives on specific areas such as evaluation and identification of babies in distress, and appropriate interventions to employ in remedying the situation. On the evaluation scale, out of a possible range of scores from zero to seven, participants had had a mean score of 4.5, with 73.8% scoring four or five. Concerning knowledge on appropriate interventions to carry out, participants had a mean score of 18.6 out of 30 with 86% scoring between 15 and 23. These findings are presented in Tables 3 and 4, respectively.

The data shows that midwives generally have insufficient knowledge on neonatal resuscitation as a whole, as well as on evaluation of babies who may need resuscitation, and how the resuscitation should be done.

### 3.3. Comparison of Knowledge in Relation to Selected Background Characteristics

Pearson correlation was used to determine association between knowledge and years of practice as a midwife, and between knowledge and previous training in neonatal resuscitation. There was no correlation between knowledge and years of practice as a midwife (p = .506). However, there was a weak positive correlation between knowledge and previous training in neonatal resuscitation that was statistically significant (*r*(158) = .195, p = .013). Details of these correlations are presented in Table 5.

A one-way ANOVA was conducted to determine differences in knowledge of participants based on their place of work and academic qualification. We found that there were statistically significant differences between groups in terms of the place of work* (F(2, 83.73) = 6.04, p = .004)*, as well as academic qualification* (F(2, 157) = 3.94, p = .021)*. The results of the ANOVA tests are presented in Table 6.

A Games-Howell post hoc test revealed that the level of knowledge among midwives in Tamale Central Hospital was statistically significantly higher* (24.67 ± 2.79, p = .014)* than midwives at Tamale West Hospital* (21.50 ± 6.24, p = .021)* and Tamale Teaching Hospital* (22.92 ± 4.56, p = .028)*. There was no statistically significant difference in the level of knowledge between those at Tamale Teaching Hospital and Tamale West Hospital (p = .418).

In terms of the academic qualification, the Games-Howell post hoc test showed that the level of knowledge among those with a first degree was statistically significantly higher* (24.34 ± 3.51, p = .027)* compared to those with a diploma. There were no statistically significant differences between those with a first degree and those with a post-NAC/NAP certificate (p = .641), and between those with a diploma and a post-NAC/NAP certificate (p = .078).

### 3.4. Experience of Midwives in Neonatal Resuscitation

The second major objective in this study was to determine the experience of midwives in neonatal resuscitation. Before presenting data about the level of experience of midwives, we first present the data about the training of participants in neonatal resuscitation, which is summarized in Table 7.

Helping Babies Breathe was the most common training in neonatal resuscitation received by participants (56.5%). It is however worrying to note that up to 19% of the participants had never undergone any training in neonatal resuscitation. The level of experience of midwives in neonatal resuscitation is summarized in Table 8. The score on the experience scale was generally low among participants. Out of a possible range of scores from zero to 435, the mean score was 30.26, and median was 15.00, with a standard deviation of 58.43. These scores placed more than half (55%) of the participants within the “not experienced” category, as displayed in Table 8.

We also compared differences in level of experience of midwives in the three different facilities using the one-way ANOVA. There were no statistically significant differences between the three groups (p = .179).

Finally, Pearson's correlation was used to measure association between experience in neonatal resuscitation and knowledge in neonatal resuscitation, and between years of practice as a midwife and experience in neonatal resuscitation. There was no correlation between experience and knowledge (*p = .793)*, or experience in neonatal resuscitation and years of practice as a midwife (*p = .506). *

## 4. Discussion

### 4.1. Knowledge of Midwives on Neonatal Resuscitation

The results from this study are actually quite worrying because it suggests that midwives are generally not knowledgeable about neonatal resuscitation, with 98.1% of participants demonstrating insufficient knowledge on the subject. This finding is consistent with the finding of Gebreegziabher, Aregawi, and Getinet [25], who studied knowledge and skills of neonatal resuscitation of health professionals at a university teaching hospital of Northwest Ethiopia and reported low levels of knowledge among midwives. It also affirms the findings of Murila, Obimbo, and Musoke [27] who reported very poor performance among health workers in Kenya (including nurses and midwives) after assessing their knowledge on neonatal resuscitation.

The finding is however contrary to the findings from a study in Afghanistan that assessed knowledge of neonatal resuscitation among doctors and midwives and reported high levels of knowledge among midwives [26]. It also contradicts findings from a study in Western Nigeria which assessed knowledge of neonatal resuscitation among nurses, and 78% of the participants demonstrated adequate knowledge in neonatal resuscitation [28].

The participants in this study also demonstrated insufficient knowledge on evaluation or assessment of babies at birth, and the appropriate interventions to carry out for babies who need resuscitation. Once again, this finding is contrary to that of Ogunlesi et al. [28], who reported that 95.5% of the participants in their study had adequate knowledge in the evaluation of babies at birth. However, the two studies conform in terms of the findings on knowledge about appropriate interventions to carry out after evaluation, with participants in both studies demonstrating inadequate knowledge in this area.

The years of practice as a midwife and the number of neonatal resuscitations performed or observed did not appear to contribute to midwives' knowledge on neonatal resuscitation. This was revealed after Person's correlations were used to test associations between these variables, and the correlations were found to be very weak and not statistically significant. This finding is contrary to that of Ogunlesi et al. [28] who observed higher levels of knowledge of neonatal resuscitation among those who practiced it before, compared to those who had not.

However, in conformity with the study of Kim et al. [26], training in neonatal resuscitation was found to be associated with higher knowledge in neonatal resuscitation, as determined by Pearson's correlation (*r*(158) = .195, p = .013). This finding suggests that there is the need to provide more training opportunities for midwives in the area of neonatal resuscitation. Also similar to the study of Kim et al., there were significant differences in the knowledge of participants based on the facility in which they practiced. Midwives at Tamale Central Hospital scored higher on knowledge scores than midwives at Tamale Teaching Hospital and Tamale West Hospital. There was no significant difference in the level of knowledge between midwives at Tamale Teaching Hospital and Tamale West Hospital. This finding is quite unexpected because, considering that Tamale Teaching Hospital is a tertiary Health Facility, it was our expectation that the participants in that facility will demonstrate a greater deal of knowledge than those at the other facilities, since they have access to more equipment and are more likely to handle complicated deliveries with babies requiring resuscitation.

Academic qualification also accounted for differences in the level of knowledge of participants. Participants with a first degree and those with a Post-NAC/NAP midwifery certificate demonstrated higher levels of knowledge than those with a diploma. There was no significant difference in the level of knowledge between those with a first degree and those with a Post-NAC/NAP certificate. This is possibly due to the fact that those with the Post-NAC/NAP certificate are usually Community Health Nurses or Enrolled Nurses with at least three years working experience before enrolling in the midwifery training program. It is therefore possible that, in their roles as Community Health Nurses or Enrolled Nurses, they were exposed to opportunities to learn about neonatal resuscitation. This finding is in sharp contrast to the findings of Murila et al. [27] who noted that nurses performed abysmally in knowledge scores regardless of whether they had a certificate or degree qualification.

### 4.2. Experience of Midwives in Neonatal Resuscitation

More than half of the participants (55%) in this study were not experienced in neonatal resuscitation. This is probably due to the relatively less experience in midwifery practice in general, since the median age of practice was 3 years. However, since there were no significant differences in the level of experience of participants based on the years of practice, it is difficult to reach this conclusion. It is however important to point out that training in neonatal resuscitation was associated with greater overall experience in the process, as determined by Pearson's correlation (*r*(158) = .196, p = .013). Unfortunately, 19% of the participants in this study had never received any training in neonatal resuscitation (Table 7). This may have contributed to the low level of experience in neonatal resuscitation among this population. There is therefore the need to provide more training opportunities for midwives

### 4.3. Conclusion

It is clear from the findings in this study that midwives generally have insufficient knowledge about neonatal resuscitation. In addition, many midwives do not have adequate experience in neonatal resuscitation, regardless of the number of years they have practiced as midwives. Midwifery training at the first degree level, basic nursing training and work experience before midwifery training, and training midwives in neonatal resuscitation may contribute to enhanced knowledge in neonatal resuscitation, since these factors were associated with higher knowledge of neonatal resuscitation. It is therefore highly imperative that government provides opportunities for all midwives to be trained in such an important lifesaving skill.

## Figures and Tables

**Table 1:** Background characteristics of participants.

**Variable**	**Frequency**	**Percentage (**%**)** **(N = 160)**
**Age group**		
20 – 24	5	3.1
25 – 29	52	32.5
30 – 34	60	37.5
35 – 39	20	12.5
40 – 44	11	6.9
45 and above	12	7.5
**Place of work**		
Tamale Teaching Hospital	79	49.4
Tamale Central Hospital	42	26.3
Tamale West Hospital	39	24.4
**Academic Qualification**		
Post NAC/NAP Certificate	79	49.4
Diploma	55	34.4
First degree	26	16.3
**Years of Practice**		
1 – 4	123	76
5 – 9	16	10
10 – 14	15	9.4
15 – 19	4	2.5
20 and above	2	1.3
**Rank**		
Staff Midwife	95	59.4
Senior Staff Midwife	21	13.1
Midwifery Officer	17	10.6
Senior Midwifery Officer	11	6.9
Principal Midwifery Officer	16	10

**Table 2:** Knowledge levels of midwives on neonatal resuscitation.

**Knowledge level**	**Frequency**	**Percentage (**%**)** **(N = 160)**
Sufficient knowledge	3	1.9
Insufficient knowledge	157	98.1

**Table 3:** Knowledge of midwives on evaluation of newborn babies.

**Knowledge level**	**Frequency**	**Percentage (**%**)** **(N = 160)**
Sufficient knowledge	21	13.1
Insufficient knowledge	139	86.9

**Table 4:** Knowledge of midwives on appropriate interventions to carry out on distressed babies.

**Knowledge level**	**Frequency**	**Percentage (**%**)** **(N = 160)**
Sufficient knowledge	3	1.9
Insufficient knowledge	157	98.1

**Table 5:** Correlations between knowledge and selected background features.

**Variables**	**Pearson *r***	**N**	**Sig. (2-tailed)**
Knowledge and years of practice	.053	160	.506
Knowledge and previous training	.195	160	.013

**Table 6:** Comparison of knowledge scores in terms of workplace and academic qualification, Welch's Anova (*N* = 160).

**Parameters**	**Sum of squares**	**Df**	**Mean square**	**F**	**Sig.**
**Workplace**					
Between groups	205.018	2	102.509	6.04	.004
Within groups	3426.246	83.73	21.823		
Total	3631.265	85.73			
**Academic Qualification **					
Between groups	180.649	2	90.324	3.68	.030
Within groups	3450.616	72.75	21.978		
Total	3631.265	79.75			

**Table 7:** Type of training received by participants in neonatal resuscitation.

**Type of training**	**Frequency**	**Percentage (**%**)**
Helping Babies Breathe	113	56.5
Essential Steps in Management of Obstetric Emergencies (ESMOE)	33	16.5
Paediatric Life Support	11	5.5
Others	5	2.5
None	38	19

**Table 8:** Experience of midwives in neonatal resuscitation.

**Experience category**	**Frequency**	**Percentage (**%**)** **(N =160)**
Not experienced	88	55.0
Moderately experienced	44	27.5
Highly experienced	28	17.5

## Data Availability

The data used to support the findings of this study have not been made available because of a confidentiality clause in our informed consent that participants signed prior to the study. Participants were made to understand that data from them will only be used for the study and will not be available to a third party.
